# Diatomite-Based Recyclable and Green Coating for Efficient Radiative Cooling

**DOI:** 10.3390/biomimetics9010050

**Published:** 2024-01-13

**Authors:** Jing Lu, Yile Fan, Xing Lou, Wei Xie, Binyuan Zhao, Han Zhou, Tongxiang Fan

**Affiliations:** 1 State Key Lab of Metal Matrix Composites, School of Materials Science and Engineering, Shanghai Jiao Tong University, Shanghai 200240, China; 120050910075@sjtu.edu.cn (J.L.); fly326@sjtu.edu.cn (Y.F.); louxing@sjtu.edu.cn (X.L.); xie--wei@sjtu.edu.cn (W.X.); byzhao@sjtu.edu.cn (B.Z.); 2 Future Materials Innovation Center, Zhangjiang Institute for Advanced Study, Shanghai Jiao Tong University, Shanghai 201203, China

**Keywords:** diatomite, radiative cooling coating, green, porous structure

## Abstract

Radiative cooling is a promising strategy to address energy challenges arising from global warming. Nevertheless, integrating optimal cooling performance with commercial applications is a considerable challenge. Here, we demonstrate a scalable and straightforward approach for fabricating green radiative cooling coating consisting of methyl cellulose matrix-random diatomites with water as a solvent. Because of the efficient scattering of the porous morphology of diatomite and the inherent absorption properties of both diatomite and cellulose, the aqueous coating exhibits an excellent solar reflectance of 94% in the range of 0.25–2.5 μm and a thermal emissivity of 0.9 in the range of 8–14 µm. During exposure to direct sunlight at noon, the obtained coating achieved a maximum subambient temperature drop of 6.1 °C on sunny days and 2.5 °C on cloudy days. Furthermore, diatomite is a naturally sourced material that requires minimal pre-processing, and our coatings can be prepared free from harmful organic compounds. Combined with cost-effectiveness and environmental friendliness, it offers a viable path for the commercial application of radiative cooling.

## 1. Introduction

Cooling to maintain suitable surface temperatures of terrestrial objects such as buildings and vehicles is now a necessity in modern society. However, traditional cooling technologies are associated with high electrical energy consumption and significant environmental impact [1]. Consequently, the adoption of innovative energy-efficient cooling designs [2] and eco-friendly solutions [3] is urgently needed. Radiative cooling, an energy-efficient and sustainable cooling approach, addresses energy and environmental concerns by reflecting solar radiation (0.25–2.5 μm) and emitting infrared radiation into outer space through the Atmospheric Transparent Spectral Window (ATSW, 8–14 μm) [4,5].

Radiative cooling is critically dependent on excellent solar reflectance, as just a few percent of solar absorbance is sufficient to counterbalance the cooling effect of infrared radiation [6]. Radiative cooling effectiveness in ideal conditions has been demonstrated by various designs, like photonic structures [7,8], flexible films [9,10] and polymer dielectric composite coatings [11,12]. Due to the combination of excellent optical properties, practicality and cost-effectiveness, radiative cooling coatings (RCCs) are superior in all designs [13]. Recent research primarily concentrates on porous structures [14] and polymer scatterer composites [15,16] to improve the solar reflectance of RCCs. Nevertheless, while attaining significant reflectivity, the strategies mentioned above commonly compromise on other key practical performance (e.g., cost, environmental impact). Specifically, the manufacturing process of porous polymer coatings typically requires a large amount of toxic organic compounds for polymer dissolution [17,18]. Remarkably, even for water-based coatings, the presence of harmful organic additives remains prevalent [13]. Such large quantities of harmful compounds not only pose environmental risks but also raise production costs. In addition, the scattering particles used in RCC can reflect most sunlight but pose a potential threat to human health (e.g., TiO_2_ [19]) and result in elevated processing costs for specific particle sizes (e.g., SiO_2_ [20]). Hence, advancing RCCs towards commercial applications, realizing excellent optical performance and simultaneously ensuring a non-toxic formula and relatively low cost remain significant challenges.

Natural bioresources have inspired the design of bio-derived radiative coolers with potential applications. For example, wood was reported to exhibit cooling effects due to the molecular vibrations of cellulose in the infrared spectrum and the high solar reflectance caused by inter-fiber channels [21]. Native silk has also been discovered to possess cooling capabilities because of the strong reflection across the visible and near-infrared spectrums induced by Anderson light localization [22]. Recently, our group discovered that the triangular fluffs of beetles [23] possess thermal regulation capabilities, as well as the microspikes of golden cicadas [24]. In addition to that, diatomite, a natural mineral formed from the remains of diatoms with a silica content exceeding 90% [20], exhibits promising potential in the field of radiative cooling. Diatomite is extensively utilized as a support substrate in phase change materials, attributable to its distinct porous structure and relative affordability [25]. However, its application in the radiative cooling field has not been substantially explored. In contrast to expensive commercial scatterers commonly used in current studies of radiative cooling, diatomite obviates the need for costly and toxic preliminary treatments, making it a cost-effective and safe alternative to artificial SiO_2_. Moreover, the surface of diatomite features nanopores comparable in size to the solar wavelengths, leading to strong Mie scattering, potentially beneficial in achieving high solar reflectance. The reserve of diatomite in China stands at ~320 million tons, with a future reserve expected to exceed 2 billion tons [26]. If these diatomite resources are fully utilized, it is not only beneficial to promote the diversified application of natural resources but also of great significance for social and economic sustainability.

In this work, we propose an aqueous RCC composed of natural diatomite, methyl cellulose and water to address both security and cost challenges. The diatom-cellulose coating is distinctively crafted using eco-friendly raw materials, entirely eschewing the use of harmful organic solvents or additives. It exhibits high reflectance across the solar region because of the hierarchical porous morphology of diatomite. A 450 µm thick hybrid coating can achieve a maximum temperature drop of 6.1 °C at noon on sunny days. Even on cloudy days with high humidity of ~60%, a maximum temperature drop of 6.5 °C across the daytime can be achieved. Furthermore, thermal cycle tests indicate that our hybrid coating exhibits promising stability against fluctuating temperatures, suggesting its suitability for extreme environmental applications. The outstanding cooling capacity and stability of the coating, along with its recyclability achieved through the use of eco-friendly solvents and natural materials, make it promising as a viable way to commercialize RCCs.

## 2. Materials and Methods

### 2.1. Design of the Diatomite-Based RCC

**Figure 1:**
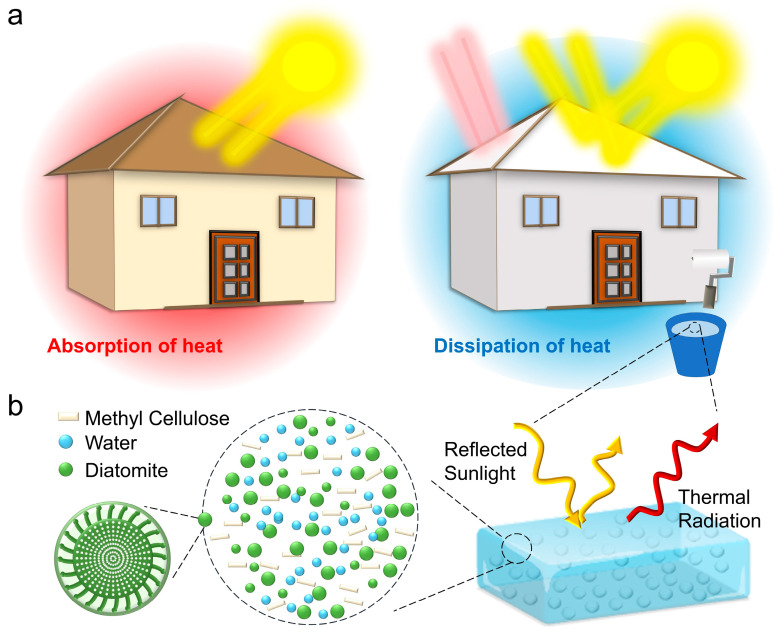
Schematic illustration of the RCC design. (**a**) The working mechanism diagram of the RCCs (on the left is the usual house, and on the right is a house painted with cooling coating). (**b**) Schematic illustration of the coating based on diatomite, methyl cellulose and water.

To effectively address the aforementioned challenges, adherence to key principles is essential. Specifically, scattering particles are supposed to avoid toxic and expensive artificial preparation processes. Simultaneously, scattering particles must possess the ability to reflect sunlight effectively. Furthermore, polymer binders can be dissolved in green solvents and mixed with scatterers without dispersants, defoamers, or other organic additives. For solar reflectivity, strong scattering induced by micro/nanoporous voids and particles introduced into the polymer matrix has developed as the conventional design principle [27,28]. Diatomite possesses a micro-nanostructure allowing effective scattering of sunlight, making it a desirable scatterer. Moreover, SiO_2_ stands out as the predominant constituent of diatomite, as depicted in Figure S1. The ideal intrinsic properties of SiO_2_ play a crucial role in ensuring elevated emissivity in the mid-infrared spectrum [9]. In addition, to avoid the use of toxic solvents, methyl cellulose was chosen as a binder, which can be dissolved directly by water. In order to eliminate bubbles caused by methyl cellulose dissolved in water, a centrifugal defoaming planetary mixer was used to obtain a uniform paint instead of dispersants and defoamers. The conceptual design of the RCC is illustrated in Figure 1, which demonstrates its application as a building surface coating.

### 2.2. Preparation of the Hybrid RCC

Diatomite was purchased from Macklin Biochemical Co., Ltd. (Shanghai, China)., and methyl cellulose was acquired from Aladdin Biochemical Technology Co., Ltd. (Shanghai, China). To remove organic impurities from the diatomite, it underwent calcination at 450 °C for 2 h [26]. Methyl cellulose was dissolved in deionized water and stirred magnetically for 4 h to achieve a uniform, transparent solution. Then, calcined diatomite powder was gradually added to the transparent solution and stirred for another 8 h to obtain a stable suspension. Methyl cellulose has a tendency to produce bubbles when dissolved in water. In order to eliminate bubbles in the suspension, it was put into the high-speed centrifugal defoaming planetary mixer, and the mixing and defoaming modes were run for 15 min at a speed of 2000 rpm, respectively. Finally, a uniform and stable paint stock solution was obtained. The paint, applied to the substrate using a brush, allows for precise control over the coating thickness by regulating the amount of paint used. The deposited coating underwent a 20 h curing process at room temperature. Alternatively, to expedite the curing process, the coating could also be placed in an oven at 40 °C and dried for 8 h. The hydrophobic coating is then obtained by spraying a hydrophobic agent (Glaco Mirror Coat Zero, Soft 99 Co., Ltd., Osaka, Japan) on the cured surface.

### 2.3. Numerical Simulations

The scattering efficiencies and electric field distributions were simulated using FDTD (Finite Difference Time Domain) Solutions (Lumerical Solutions Inc., Vancouver, BC, Canada). SiO_2_ was used as the material for the simulated models. The TFSF (Total-Field Scattered-Field) source was employed in our simulations to effectively separate the incident and scattered fields, which is crucial for accurate scattering analysis. The PML (Perfectly Matched Layer) boundary conditions were applied to all edges of the simulation domain to mimic an unbounded space. The source wavelengths were set at 0.25–2.5 µm, with incident propagation along the negative z-axis and y-axis. The scattering efficiencies were computed by dividing the scattering cross-section by the geometric shadow area, based on cross-section analysis units. To explore the impact of diatomite’s porous structure, the electric field distributions were monitored using frequency-domain field and power monitors. More details can be found in the Supplementary Materials (Table S1).

### 2.4. Optical Characterizations

The reflectance of the diatomite-based hybrid RCCs in the solar region ($R_{\mathrm{solar}}$) were measured using the UV/VIS/NIR (ultraviolet–visible–near infrared) spectrophotometer (Lambda 950, PerkinElmer Inc., Waltham, MA, USA) with an integrating sphere setup. The transmittance and reflectance spectra of the hybrid RCCs within the atmospheric transparent window region (8–14 μm) were obtained using a Fourier transform infrared spectrometer (Nicolet 6700, Thermo Fisher Scientific Inc., Waltham, MA, USA) equipped with an infrared integrating sphere. The emissivity/absorptivity of the coatings were then calculated as 1—Reflectance—Transmittance. Since the transmittance of all samples is nearly zero, the emissivity spectra in the atmospheric window can essentially be determined by 1—Reflectance.

### 2.5. Calculation of $R_{\mathrm{solar}}$ and $\varepsilon_{\mathrm{ATSW}}$

The $R_{\mathrm{solar}}$ of the coating is calculated as follows:

(1) $$R_{\mathrm{solar}}=\frac{\int_{0.25}^{2.5}I_{\mathrm{AM}1.5}(\lambda )R(\lambda )d\lambda }{\int_{0.25}^{2.5}I_{\mathrm{AM}1.5}(\lambda )d\lambda }$$

Here, $I_{\mathrm{AM}1.5}\left(\lambda \right)$ represents the ASTM G173 solar spectra, while R(λ) denotes the reflectance of the diatom-cellulose hybrid RCCs.

Similarly, the emissivity in the ATSW ($\varepsilon_{\mathrm{ATSW}}$) is calculated as follows:

(2) $$\varepsilon_{\mathrm{ATSW}}=\frac{\int_{8}^{14}I_{\mathrm{BB}}(T,\lambda )\varepsilon (\lambda )d\lambda }{\int_{8}^{14}I_{\mathrm{BB}}(T,\lambda )d\lambda }$$

Here, $I_{\mathrm{BB}}(T,\lambda )$ is the radiation emitted by a black body at the temperature of T according to Planck’s law, and ε(λ) is the spectral emittance of the coating.

### 2.6. Outdoor Measurement

To assess the real cooling efficiency of the RCCs, self-developing experimental equipment was utilized. As shown in Figure S2, the skeleton of the device was constructed from K9 glass due to its high transparency in the visible and near-infrared spectra, minimizing interference with the sample. The middle part of the K9 glass was hollowed out and replaced with LDPE membrane to hold the samples. Plastic foam was adhered to both the upper and lower surfaces of the K9 glass, and an outer layer of LDPE film was wrapped around the plastic foam to minimize heat transfer between the samples and the surrounding air. The LDPE film exhibited high transparency across the visible and mid-infrared regions. Consequently, neither the coating’s absorption of solar heat nor its radiative cooling capabilities were affected. A thermocouple placed against the back of the samples was used to measure their temperature, while the ambient temperature was recorded using a weather louver. To avoid the interference of the black floor in the sample temperature, we suspended the experimental device on the ground with a PET/aluminum alloy bracket higher than 1 m.

### 2.7. Net Cooling Power Calculation

In order to understand the cooling potential of the diatom-cellulose hybrid RCC more intuitively, we calculated the theoretical net cooling power ($P_{\mathrm{net}}$) of the RCC. It is represented by Equation (3) below [10]:

(3) $$P_{\mathrm{net}}=P_{\mathrm{rad}}-P_{\mathrm{sun}}-P_{\mathrm{atm}}-P_{\mathrm{con}}$$

where $P_{\mathrm{rad}}$ represents the thermal radiation power of the coating, as shown in Equation (4):

(4) $$P_{\mathrm{rad}}=2\pi \int_{0}^{\pi /2}\sin\theta \cos\theta d\theta \int_{0}^{\infty }I_{\mathrm{BB}}(T_{r},\lambda )\varepsilon_{r}\left(\theta ,\lambda \right)d\lambda$$

Here, $T_{r}$ is the temperature of the RCC, $\varepsilon_{r}\left(\theta ,\lambda \right)\;$ represents the spectral emissivity of the RCCs at angle θ and wavelength λ.

$P_{\mathrm{sun}}$ represents the solar radiation power absorbed by the RCC, as defined in Equation (5):

(5) $$P_{\mathrm{sun}}=\int_{0}^{\infty }I_{\mathrm{AM}1.5}\left(\lambda \right)\varepsilon_{r}\left(\lambda ,\theta_{\mathrm{sun}}\right)d\lambda$$

Here, $\varepsilon_{r}\left(\lambda ,\theta_{\mathrm{sun}}\right)$ represents the absorptivity of the RCC at angle $\theta_{\mathrm{sun}}$ and wavelength λ.

$P_{\mathrm{atm}}$ represents the thermal radiation from atmosphere absorbed by the coating and is calculated using Equation (6):

(6) $$P_{\mathrm{atm}}=2\pi \int_{0}^{\pi /2}\sin\theta \cos\theta d\theta \int_{0}^{\infty }I_{\mathrm{BB}}(T_{\mathrm{amb}},\lambda )\varepsilon_{r}\left(\theta ,\lambda \right)\varepsilon_{\mathrm{atm}}\left(\theta ,\lambda \right)d\lambda$$

Here, $T_{\mathrm{amb}}$ is the temperature of ambient, $\varepsilon_{\mathrm{atm}}\left(\theta ,\lambda \right)$ is the emittance of atmosphere and can be expressed as $\varepsilon_{\mathrm{atm}}\left(\theta ,\lambda \right)=1-{t(\lambda )}^{1/\cos\theta }$, where t(λ) represents the transparency of the atmosphere.

$P_{\mathrm{con}}$ represents the non-radiative heat exchange power, calculated using Equation (7):

(7) $$P_{\mathrm{con}}=\left(T_{\mathrm{amb}}-T_{r}\right)\times q$$

Here, q represents the composite heat conduction and convection exchange coefficient of the RCC with ambient air, and it can generally be limited to the range between 2 and 6.9 Wm^−2^K^−1^ [29].

## 3. Results and Discussion

### 3.1. Optical Properties of the Materials

**Figure 2:**
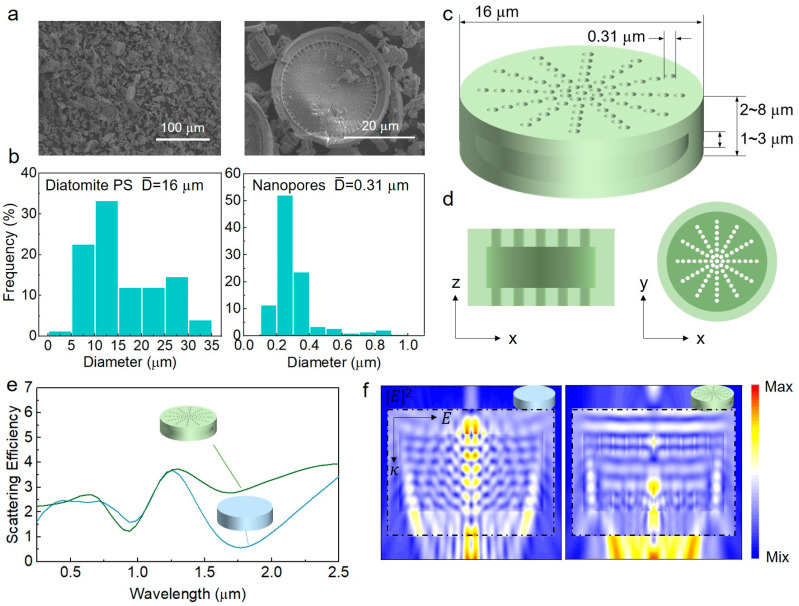
Morphology and high reflectance characteristics of diatomite. (**a**) The SEM images of the diatomite. (**b**) Particle size (PS, left) and pore size (right) distribution of the diatomite. (**c**) Structure diagram and (**d**) cross-section diagrams of the diatomite. (**e**) Scattering efficiency of the diatomite structure (green) and pure SiO_2_ structure (blue) in solar region (0.25–2.5 μm) with perpendicular incident light. (**f**) The electric field distributions of the diatomite structure (right) and pure SiO_2_ structure (left) at ~1.7 μm wavelength (the black line indicates the TFSF source region, with the area outside representing the scattered field induced by the TFSF source).

As shown in Figure 2a, the diatomite surface exhibits a porous structure. Pore-size analysis reveals that the particle and pore sizes of the diatomite are broadly distributed, with average values ($\bar{D}$) of ~16 and ~0.3 μm (Figure 2b–d), respectively. FDTD simulations show that the particles with a diameter of ~16 μm effectively scatter sunlight across the entire wavelength range from 0.25 to 2.5 μm (Figure 2e and Figure S3). When the incident light is perpendicular (Figure 2e), the diatomite structure exhibits higher scattering efficiency in the near-infrared spectrum compared to the SiO_2_ model without a porous structure, suggesting that the porous nature of diatomite further enhances scattering. This is corroborated by the electric field distributions in the near-infrared region. As shown in Figure 2f, the diatomite with a porous structure exhibits stronger scattering capability in the scattered field. Considering the scenario of horizontal incidence of light, both models exhibit essentially the same high scattering efficiency in the solar spectrum (Figure S3). This suggests that diatomite, with a particle size of 16 µm and a surface with ~300 nm nanopores, is an effective scatterer across the entire solar spectrum. On the one hand, the pore sizes of the diatomite surface are widely distributed in the ultraviolet to near-infrared wavelength range, which can lead to strong Mie scattering of incident solar radiation. On the other hand, the scattered solar radiation is not absorbed by the coating due to the lossless properties of silica and the polymer matrix within the solar region [9,21]. Consequently, this results in high reflectance of the RCC in the range of 0.25–2.5 μm. In addition, according to the Fourier transform infrared transmission spectrum (Figure S4), the symmetric stretching vibration peaks of Si-O bonds (800 cm^−1^) and the anti-symmetric stretching vibration peaks of Si-O-Si bonds (1090 cm^−1^) of diatomite fall precisely within the ATSW (8–14 µm), resulting in strong emissive characteristics in this region. Meanwhile, methyl cellulose exhibits multiple absorption peaks in the ATSW (Figure S5), enhancing the emissivity in this region.

### 3.2. Morphology and Optical Performance of the RCCs

**Figure 3:**
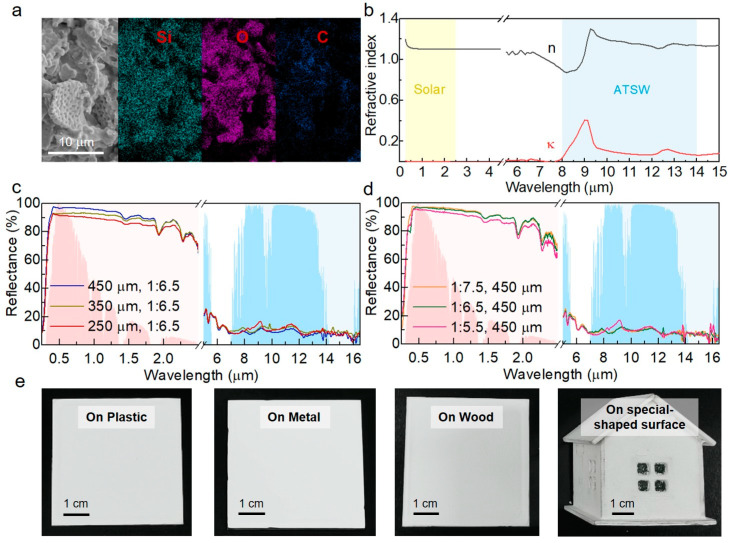
Morphology and optical characterization of the hybrid RCC. (**a**) SEM and EDS mapping images of the diatom-cellulose hybrid RCC. (**b**) The refractive index (n) and extinction coefficient (k) of the diatom-cellulose hybrid RCC. (**c**) Reflectance spectra of the RCCs in the solar and ATSW range at different thicknesses. (**d**) Reflectance spectra of the RCCs in the solar and ATSW range at different mass ratios of binder-to-filler (methyl cellulose to diatomite). (**e**) The optical images of the 450 µm thick coating applied on different surfaces.

We developed an aqueous RCC that incorporates diatomite particles randomly dispersed within a methyl cellulose matrix. The fabrication process is straightforward, green and potentially scalable. As shown in Figure 3a, the microstructure of the diatom-cellulose hybrid coating still exhibits a porous morphology. The complex refractive index of the coating (Figure 3b) indicates that within the solar range of 0.25~2.5 μm, the extinction coefficient (k) of the coating approaches zero and can be considered negligible, ensuring minimal solar energy absorption. Furthermore, the coating exhibited a distinct extinction peak in the ATSW, enabling effective heat radiation in that region. Figure 3c,d illustrate the reflective properties of the coatings across the solar and LWIR wavelengths, with AM 1.5G solar radiation (pink) and atmospheric transmittance (blue) in the background. At a thickness of 450 µm, the diatom-cellulose hybrid coating exhibited an excellent reflectivity of ~94% in the solar range and a high emissivity of ~0.9 in the ATSW.

The thickness of the coating is a crucial factor in determining the $R_{\mathrm{solar}}$ and $\varepsilon_{\mathrm{ATSW}}$. As illustrated in Figure 3c, both $R_{\mathrm{solar}}$ and $\varepsilon_{\mathrm{ATSW}}$ (ε(λ) = 1 − R(λ)) of the coating increase with thickness, with the rise in $R_{\mathrm{solar}}$ being more significant than that in $\varepsilon_{\mathrm{ATSW}}$ as the thickness increases. However, in the near-infrared band, the reflectance variations gradually diminish, suggesting a limited benefit in reflectance improvement with further increases in thickness. Additionally, an important experimental observation was that coatings exceeding a certain thickness tended to crack, which poses practical limitations. Consequently, a coating thickness of 450 μm was selected based on considerations of cost-effectiveness and practicality. Moreover, the effects of the mass ratio of the binder-to-filler (methyl cellulose to diatomite) in the coating on the optical performance were also investigated. As illustrated in Figure 3d, the $R_{\mathrm{solar}}$ and $\varepsilon_{\mathrm{ATSW}}$ of the diatom-cellulose hybrid RCC increased with the rising concentration of diatomite. Remarkably, both properties remained relatively stable once the diatomite concentration reached a certain threshold (methyl cellulose:diatomite = 1:6.5). The porous diatomite particles distributed in the coating enhanced scattering across the ultraviolet to visible spectrum, which contributed to the high $R_{\mathrm{solar}}$ of the coating. However, for practical applications, challenges like coatings cracking often exist, especially when the filler concentration is excessively high. To keep a balance between practicality and optimal performance, the optimal ratio of methyl cellulose to diatomite was determined as 1:6.5. The delicate balance ensures the stability and high performance of the hybrid coating simultaneously, resulting in the coating achieving a $R_{\mathrm{solar}}$ of 94% and a $\varepsilon_{\mathrm{ATSW}}$ of 0.9. Additionally, due to the nature of room temperature and liquid-based processes, the diatom-cellulose hybrid RCC can be applied on various surfaces, including metal, wood, plastic and other irregularly shaped surfaces, using conventional methods such as brushing (Figure 3e).

### 3.3. Cooling Performance of the RCCs

**Figure 4:**
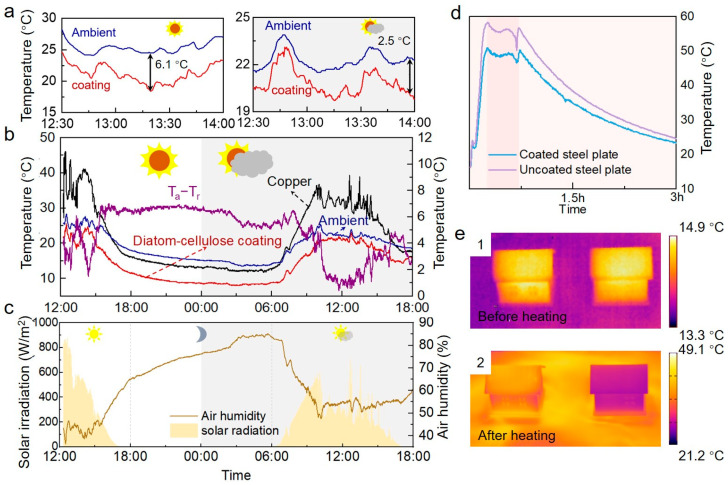
The cooling performance of the RCCs. (**a**) Cooling performance of the diatom-cellulose hybrid coating at noon. (**b**) Cooling performance of the coating under sunny and cloudy conditions. (**c**) Solar radiation and air humidity on sunny and cloudy days, respectively. (**d**) Temperature comparison of coated and uncoated steel plates during and after heating test. (**e**) Comparison of the model coated with the cooling coating (right) and the naked model (left) before and after heating for a period of time in the infrared camera.

To investigate the cooling efficiency of the aqueous coating developed above, an outdoor experiment was conducted in Shanghai, China, on 2–3 November 2022. Although cooling requirements seem to be reduced during winter, effective cooling remains crucial, especially for buildings with large electrical equipment. We measured the cooling efficiency both in sunny conditions and cloudy days. Figure 4a,b demonstrate that the temperature of our coating remained consistently lower than the ambient temperature (indicated by the purple line, where T_a_ represents ambient temperature and T_r_ represents the RCC’s temperature), regardless of sunny or cloudy conditions. On sunny days, the coating achieved a considerable reduction in temperature, showing a peak drop of 6.1 °C and an average decrease of over 4 °C around midday when the sun is strongest, as illustrated in Figure 4a. During the daytime, it decreased by up to 7 °C (Figure S6). On cloudy days, the coating still exhibited pronounced radiative cooling, achieving a peak temperature drop of 2.5 °C during midday (Figure 4a) and cooling down by up to 6.5 °C throughout the daytime (Figure S6). Furthermore, when the aqueous coating was applied to a metal surface, it exhibited a temperature decrease of up to 31 °C compared to the bare substrate (Figure S7). The outdoor experiment was carried out in a coastal city in winter, where the ambient humidity was relatively high. During sunny days, the relative humidity at noon was approximately 40%, and it rose to over 80% at night. On cloudy days, the relative humidity remained close to 60% during midday (Figure 4c). High humidity raises atmospheric radiation and lowers atmospheric transmittance, increasing heat absorption by the coating and impeding effective heat radiation into space, thereby diminishing radiative cooling efficiency [30]. Despite this, our coating still achieved an excellent cooling performance, which proves that it has outstanding stability and practicality. To evaluate the cooling effect of our coating under high-temperature conditions, we conducted an experiment where both a bare steel plate and a steel plate coated with diatomite-based coating were placed on a heating platform set to nearly 60 °C. We monitored the temperature changes of both plates over a period, as illustrated in Figure 4d. Consistently, the temperature of the coated plate remained lower than that of the uncoated plate. At thermal steady state, the coated plate’s temperature was on average 7 °C lower than the uncoated plate. Furthermore, the cooling efficiency of the hybrid coating was confirmed through the infrared camera, which is illustrated in Figure 4e. The naked wooden house model and the one coated with the hybrid coating were both heated at 60 °C for a period of time. Here, the wooden model was chosen for comparison because of its inherent radiative cooling capabilities [21]. As depicted in Figure 4(e1), the temperatures of both models were similar prior to heating. Upon reaching thermal equilibrium, Figure 4(e2) demonstrates a notable temperature reduction in the coated model compared to the naked house model.

**Figure 5:**
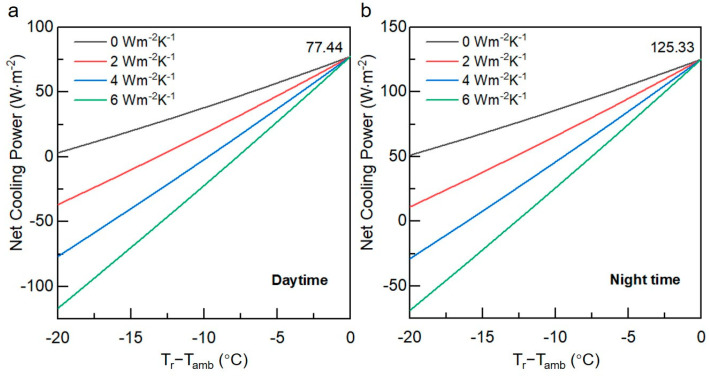
The net cooling power of the diatom-cellulose hybrid RCC. During the daytime (**a**) and nighttime (**b**) with different non-radiative heat exchange coefficients.

Moreover, the cooling capacity of the diatom-cellulose hybrid RCC was calculated. Figure 5a shows the results at different non-radiative heat exchange combined coefficients q during the daytime, while Figure 5b displays the results during the nighttime. During the daytime, the theoretical radiative cooling power of the RCC could reach 77.44 Wm^−2^ at thermal equilibrium, given an ambient temperature of 298 K. This value increases to 125.33 Wm^−2^ at night. This implies that the diatom-cellulose hybrid RCC exhibits exceptional cooling performance throughout the day and night. The higher cooling power at night is because objects do not absorb energy from the sun during the nighttime but still emit thermal radiation outward. Figure 5 reveals that the net cooling power is significantly affected by q, with the highest theoretical temperature drop reaching ~33 °C at night and ~21 °C during daytime when q = 0 Wm^−2^K^−1^. When q = 6 Wm^−2^K^−1^, these values decrease to 13 °C and 8 °C, respectively. The calculated values are higher than those of outdoor experiments due to factors such as air humidity affecting the actual measurements. Furthermore, according to the results shown in Figure 5, if the test setup can be improved to reduce non-radiative heat exchange, namely thermal convection and conduction [31], the coating would exhibit even better cooling performance.

### 3.4. Utility and Stability of the RCCs

**Figure 6:**
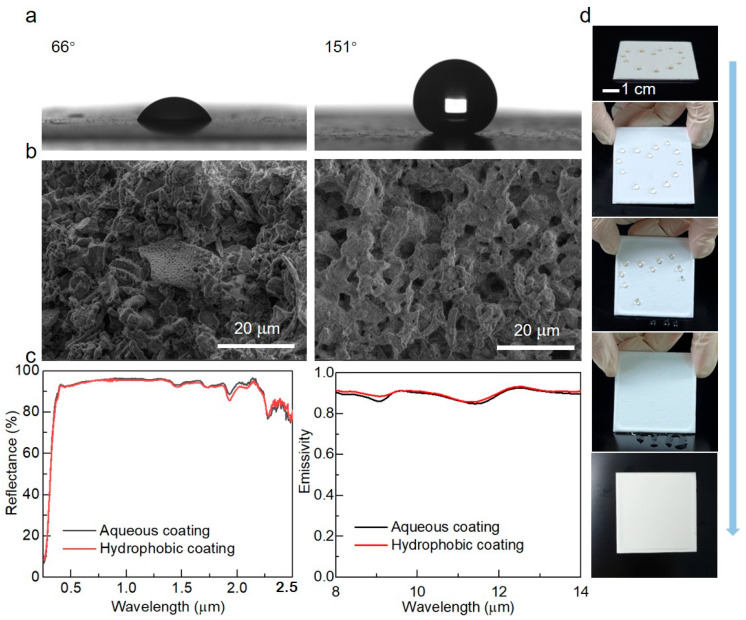
Utility of the hybrid RCCs. (**a**) Contact angle of the aqueous diatom-cellulose hybrid coating (**left**) and the one sprayed with the hydrophobic agent (**right**). (**b**) The SEM image of the aqueous coating (**left**) and the hydrophobic coating (**right**). (**c**) $R_{\mathrm{solar}}$ and $\varepsilon_{\mathrm{ATSW}}$ of the aqueous coating and hydrophobic one. (**d**) The hydrophobic performance of the coating sprayed with the agent.

In practical applications, natural substances like dust and water vapor can contaminate the aqueous coating surface, thereby affecting the cooling efficiency. To enhance the practicality outdoors, a thin layer of hydrophobic agent was applied to the surface of the diatomite-cellulose hybrid RCC. As shown in Figure 6a, it is obvious that the contact angle of the coating significantly increases to over 150° after hydrophobic treatment. Notably, the coating surface maintains its porous microstructure even after hydrophobic treatment (Figure 6b), leading to the hydrophobic coating retaining $R_{\mathrm{solar}}$ and $\varepsilon_{\mathrm{ATSW}}$ consistent with the original aqueous coating (Figure 6c). Clearly, the particles in the hydrophobic agent merely coated the surface of the aqueous coating with a thin layer, without altering its original optical performance. To further investigate the self-cleaning performance, we conducted an experiment by placing a water-based colored solution on the coating surface. It was observed that the solution hardly wetted the coating surface (Figure 6d), indicating its excellent self-cleaning properties.

**Figure 7:**
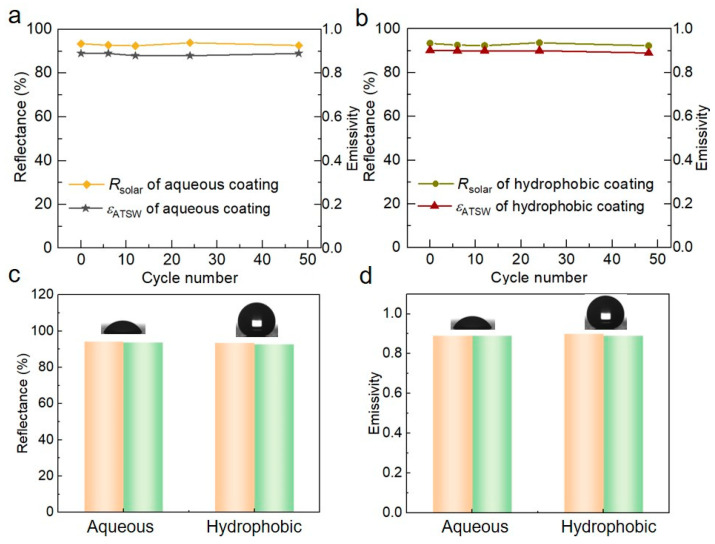
Stability of the hybrid RCCs. (**a**) The optical performance of the aqueous and (**b**) hydrophobic coatings in the thermal cycling tests. (**c**) The $R_{\mathrm{solar}}$ of the aqueous and hydrophobic coatings before (orange) and after (green) UV accelerated aging test and dry–wet cyclic exposure. (**d**) The $\varepsilon_{\mathrm{ATSW}}$ of the aqueous and hydrophobic coatings before (orange) and after (green) UV accelerated aging test and dry–wet cyclic exposure.

Moreover, a series of comprehensive assessments were conducted to explore the stability and durability of the obtained aqueous/hydrophobic RCCs, including thermal cycling tests, thermogravimetric analysis (TGA) and accelerated aging tests. As illustrated in Figure 7a,b, the optical properties of the hydrophobic and aqueous RCCs remained stable after 48 thermal cycles (0–100 °C, Figure S8), indicating that our RCCs maintained good performance stability even under extreme heat conditions. Meanwhile, TGA analysis (Figure S9) demonstrates that both the aqueous coating and the hydrophobic coating maintain thermal stability below 300 °C. Additionally, as displayed in Figure 7c,d, the UV accelerated aging test combined with dry–wet cyclic exposure was conducted to observe the weather resistance of the RCCs. The optical characteristics of the coatings remained virtually unchanged after the test (Figure S10), indicating the exceptional durability and practicality of the RCCs.

### 3.5. Performance Comparison of the Diatomite-Based Coating with Other Works

The comparative summary of the diatomite coating’s solar reflectance, midday cooling performance, thickness and environmental friendliness with other notable works is presented in Table 1. The reason for selecting midday as the observation period is due to the greater difficulty of cooling under direct sunlight at noon, which makes the cooling capacity during this time most indicative of the material’s effectiveness. Table 1 reveals that our coating is on par with advanced designs, achieving substantial reflectivity and cooling efficiency with a relatively thinner layer. Furthermore, our coating is formulated without harmful raw materials and is cost-effective (Table S2) [12,28,31], offering significant environmental and cost advantages.

## 4. Conclusions

Drawing inspiration from the natural source of diatomite, we processed an eco-friendly RCC without any harmful organic compounds. Influenced synergistically by the hierarchical pores of diatomite and the intrinsic properties of both diatomite and methyl cellulose, the coating achieves remarkable performance with $R_{\mathrm{solar}}$ of 94% and $\varepsilon_{\mathrm{ATSW}}$ of 0.9. Such characteristics result in a subambient temperature reduction of 7 °C during sunny days and 6.5 °C on cloudy days. Furthermore, the coating offers remarkable benefits including exceptional adaptability to various substrates, impressive thermal stability and absolute non-toxicity. Notably, it exhibits effective cooling performance under both sunny and cloudy weather conditions, as well as in normal and higher temperature environments, demonstrating its broad applicability. More importantly, diatomite is a widely accessible and cost-effective natural resource. This work substantially broadens the scope of diatomite’s applications, fostering sustainable methodologies and improving resource utilization efficiency. In conclusion, the developed coating offers a pathway for implementing bioinspired approaches in commercial radiative cooling applications.

## Figures and Tables

**Table 1:** Comparative analysis of diatomite-based coating with other designs in radiative cooling.

Author	Material	Thickness (μm)	$R_{\mathrm{solar}}$ (%)	Testing Time	Temperature Drop (°C)	Use of Unsafe Solvent/Additive
This work	Diatomite/Methyl cellulose	450	94	~12:00–14:00	6.1 (Max) 4.1 (Avg)	None
Song et al. [4]	PVDF/PVDF–HFP	2 mm	96	~12:00	2.2	Acetone
Huang et al. [13]	P(VdF-HFP)	500	94	12:30–14:00	1.7 ^1^ (Avg)	Sodium dodecyl sulfonate
Du et al. [16]	Y_2_O_3_/TiO_2_/PDMS	150	92.2	10:00–14:00	7.7 (Avg)	N-hexane
Li et al. [21]	Wood	/	97 (400–700 nm)	11:00–14:00	>4 (Avg)	H_2_O_2_
Chen et al. [32]	SiO_2_/Cellulose	4 mm	94	noon	6	Hydrofluoric acid
Liu et al. [31]	BaSO_4_/Ethyl cellulose	700	98.6	noon	>2.5	Ethanol

^1^ Value without convection shield.

## Data Availability

Raw data for this work may be obtained from the corresponding authors upon reasonable request.

## Supplementary Materials

The following supporting information can be downloaded at: https://www.mdpi.com/article/10.3390/biomimetics9010050/s1, Figure S1: X-ray Diffraction and Energy Dispersive Spectroscopy analysis of the calcined diatomite; Figure S2: The test equipment in the outdoor experiment; Figure S3: Scattering efficiency of the diatomite structure (green) and pure SiO_2_ structure (blue) in solar region (0.25–2.5 μm) with parallel incident light; Figure S4: The FTIR spectra of the diatomite; Figure S5: The FTIR spectra of the methyl cellulose; Figure S6: The temperature records of radiative cooling coating and ambient; Figure S7: The outdoor tests of the radiative cooling coatings and bare substrates; Figure S8: Performance variations of the coatings under the thermal cycles (0–100 °C) for over 220 h; Figure S9: The thermogravimetric analysis of the aqueous coating and hydrophobic coating; Figure S10: The performance of the coatings before/after the UV accelerated aging test combined with dry–wet cyclic exposure. Table S1: Summary of relevant simulations parameters; Table S2: Cost comparison of diatomite-based solution with other radiative cooling materials.
