# Supplementary material for: Diatomite-Based Recyclable and Green Coating for Efficient Radiative Cooling
Source: Biomimetics (Basel). 2024 Jan 13;9(1):50. doi: 10.3390/biomimetics9010050 (PMC10813072; doi:10.3390/biomimetics9010050)
Supplement: Supplementary file 1 [file biomimetics-09-00050-s001.zip › biomimetics-2739016-supplementary.pdf]

Diatomite-based recyclable and green coating for efficient radiative cooling

Jing Lu[1], *Yile Fan*[1], *Xing Lou*[1], *Wei Xie*[1], *Binyuan Zhao*[1], *Han Zhou*[1,2]*, *Tongxiang Fan*[1,]*

[1]State Key Lab of Metal Matrix Composites, School of Materials Science and Engineering, Shanghai Jiao Tong University, Shanghai 200240, China

E-mail: hanzhou_81@sjtu.edu.cn, txfan@sjtu.edu.cn

[2]Future Materials Innovation Center, Zhangjiang Institute for Advanced Study, Shanghai Jiao Tong University, Shanghai, 201203, China

Composition analysis of diatomite

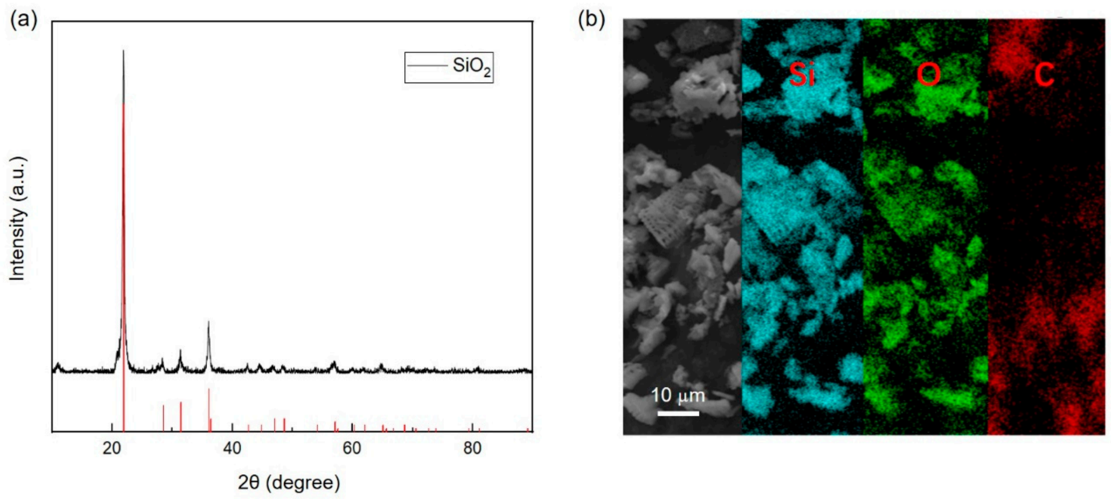

Figure S1. X-ray diffraction (a) and energy dispersive spectroscopy (b) analysis of the calcined diatomite.

Software simulation parameters

Table S1. Summary of relevant simulations parameters.

Parameter	Value
Model size	Diameter: 16 μm Height: 4 μm
Material parameter	SiO_2 (refractive index (n, k) values taken from the FDTD software material database)
Boundary conditions	PML (Perfectly Matched Layer)
Source	TFSF (Total-Field Scattered-Field)
Simulation time	44 hrs, 35 mins, 5 secs.
Mesh accuracy	3
Early shutoff	1E-6

Outdoor test equipment

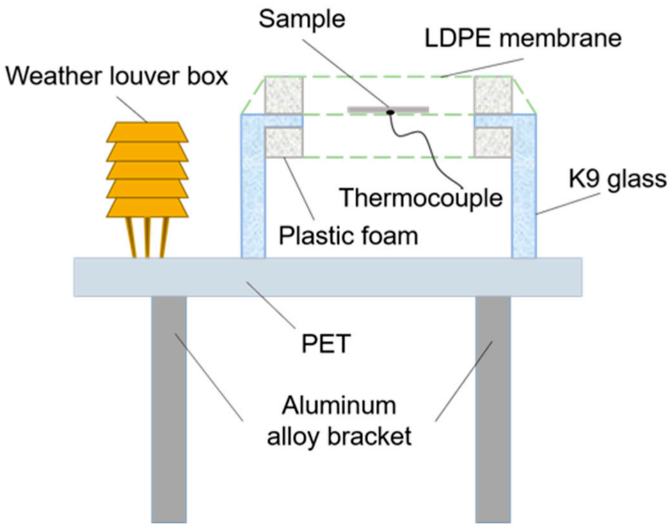

Figure S2. The test equipment in the outdoor experiment.

Scattering efficiency under horizontal sunlight incidence

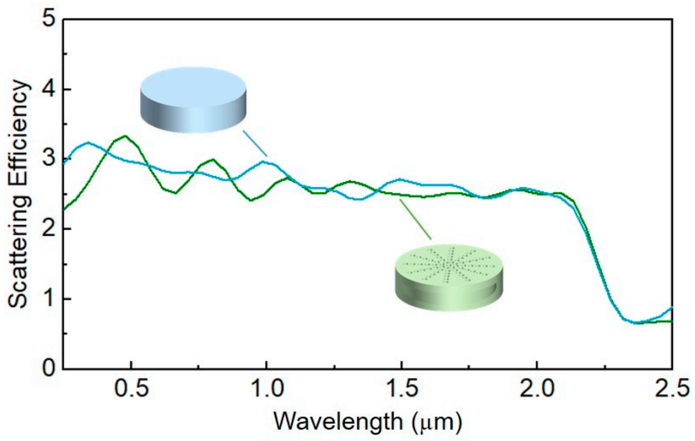

Figure S3. Scattering efficiency of the diatomite structure (green) and pure SiO_2 structure (blue) in solar region (0.25-2.5 μm) with parallel incident light.

When the incident light is parallel, the scattering efficiency of both the diatomite and pure SiO_2 structures is shown in Fig. S3. In the solar spectrum (0.25-2.5 μm), both models demonstrate high scattering efficiency. Since the structures of both models are virtually identical in the X-Z/Y-Z views for the size range close to solar wavelengths, they exhibit essentially the same scattering trends. The scattering efficiency, derived from dividing the scattering cross-section by the geometric shadow area, reveals diatomite's effectiveness as a scatterer in enhancing solar spectrum scattering.

FTIR spectra of the diatomite and methyl cellulose

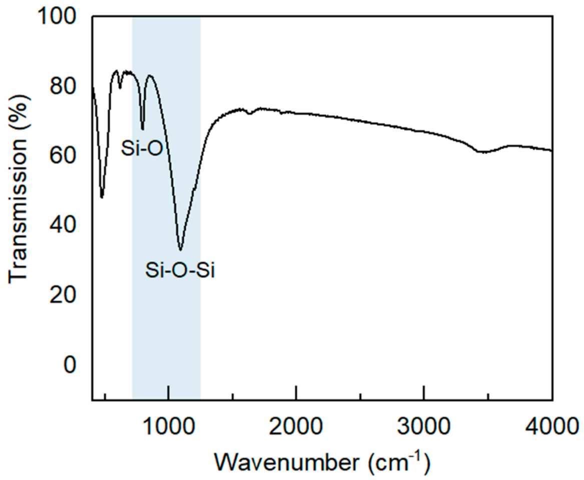

Figure S4. The FTIR spectra of the diatomite.

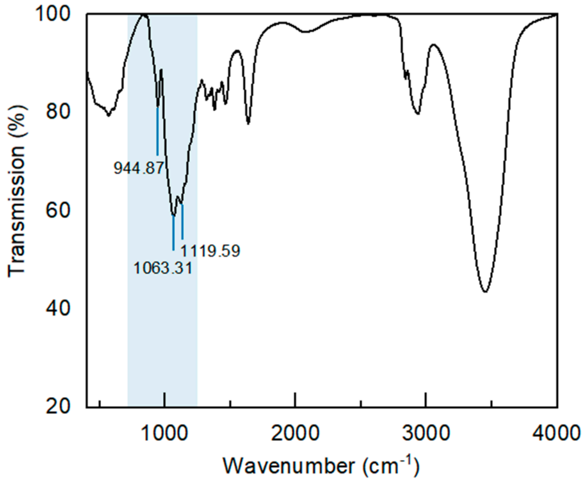

Figure S5. The FTIR spectra of the methyl cellulose.

As shown in Fig. S5, methyl cellulose has multiple absorption peaks in the ATSW, which ensures high emissivity of the hybrid coating in the region.

The outdoor test

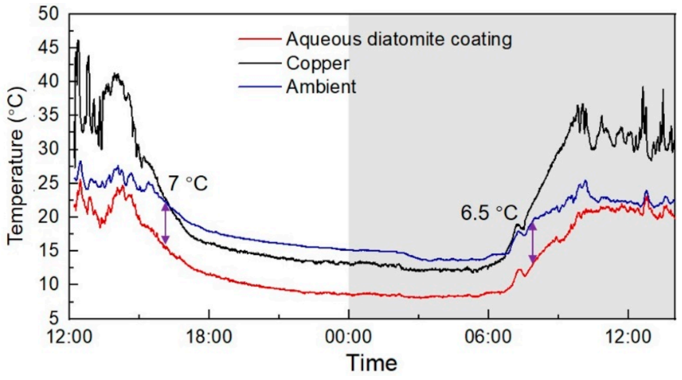

Figure S6. The temperature records of radiative cooling coating and ambient (November 2nd-3rd, 2022, in Shanghai, China.).

On sunny days, our radiative cooling coating achieved a maximum temperature reduction of 7 °C below the ambient temperature. On cloudy days, the coating lowered the temperature by 6.5 °C below the ambient level.

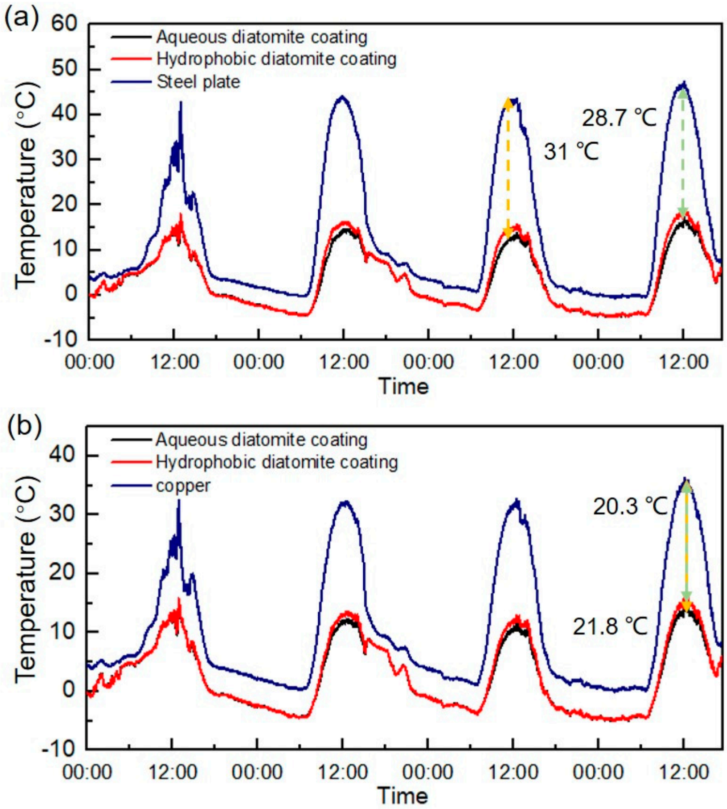

Figure S7. The outdoor tests of the radiative cooling coatings and bare substrates (December 11th-14th, 2022, in Shanghai, China.). a) The temperature records of bare steel and steel covered with radiative cooling coatings. b) The temperature records of bare copper and copper covered with radiative cooling coatings.

Another outdoor experiment was carried out on December 11th-14th, 2022, in Shanghai, China. In this experiment, we compared the temperature changes of steel and copper plates covered with aqueous/hydrophobic radiative cooling coatings with bare plates under direct sunlight. The steel covered with the aqueous radiative cooling coating can achieve a maximum temperature drop of 31 °C, and the copper covered with the aqueous coating can achieve a maximum temperature drop of 21.8 °C.

Thermal cycling tests

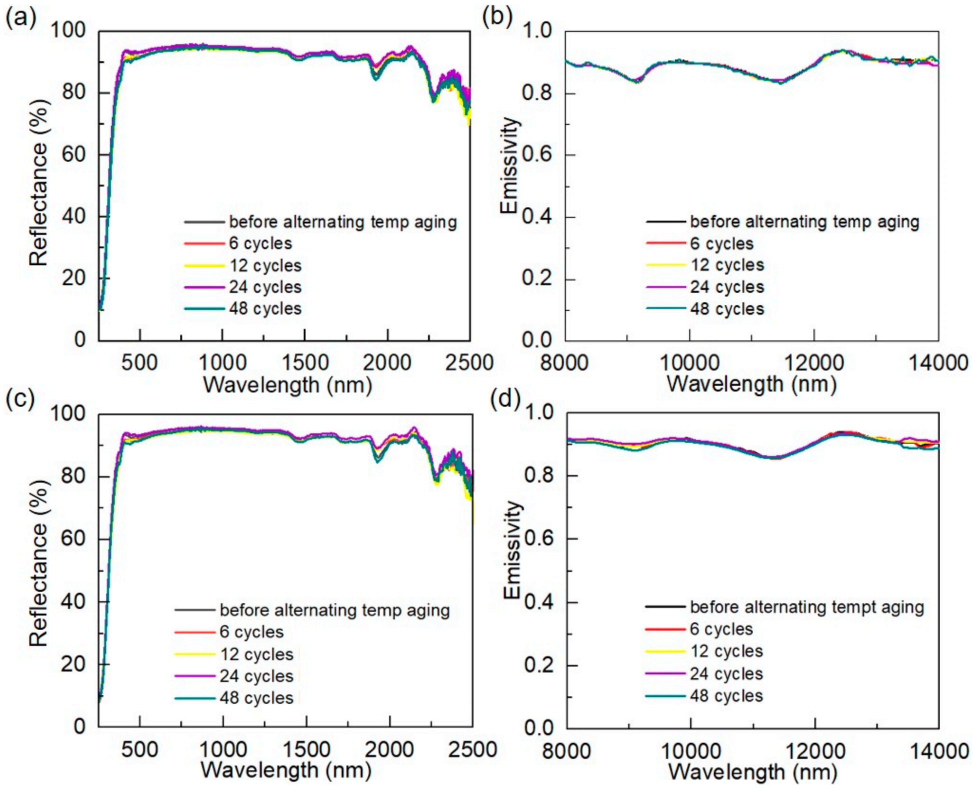

Figure S8. Performance variations of the coatings under the thermal cycles (0-100°C) for over 220h. The reflectance a) and emissivity b) of the aqueous coating. The reflectance c) and emissivity d) of the hydrophobic coating.

Thermogravimetric analysis

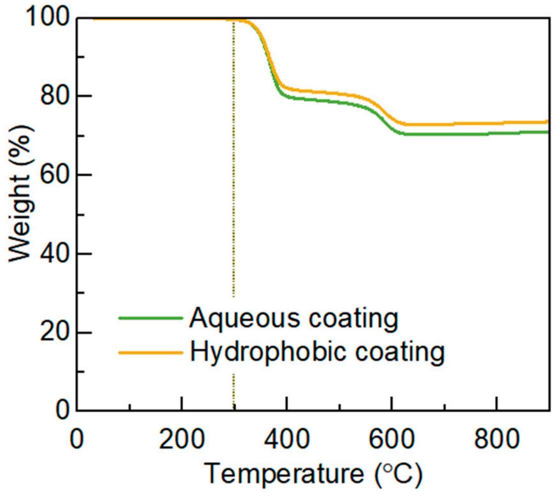

Figure S9. The thermogravimetric analysis of the aqueous coating and hydrophobic coating.

Ultraviolet accelerated aging test and dry–wet cyclic exposure

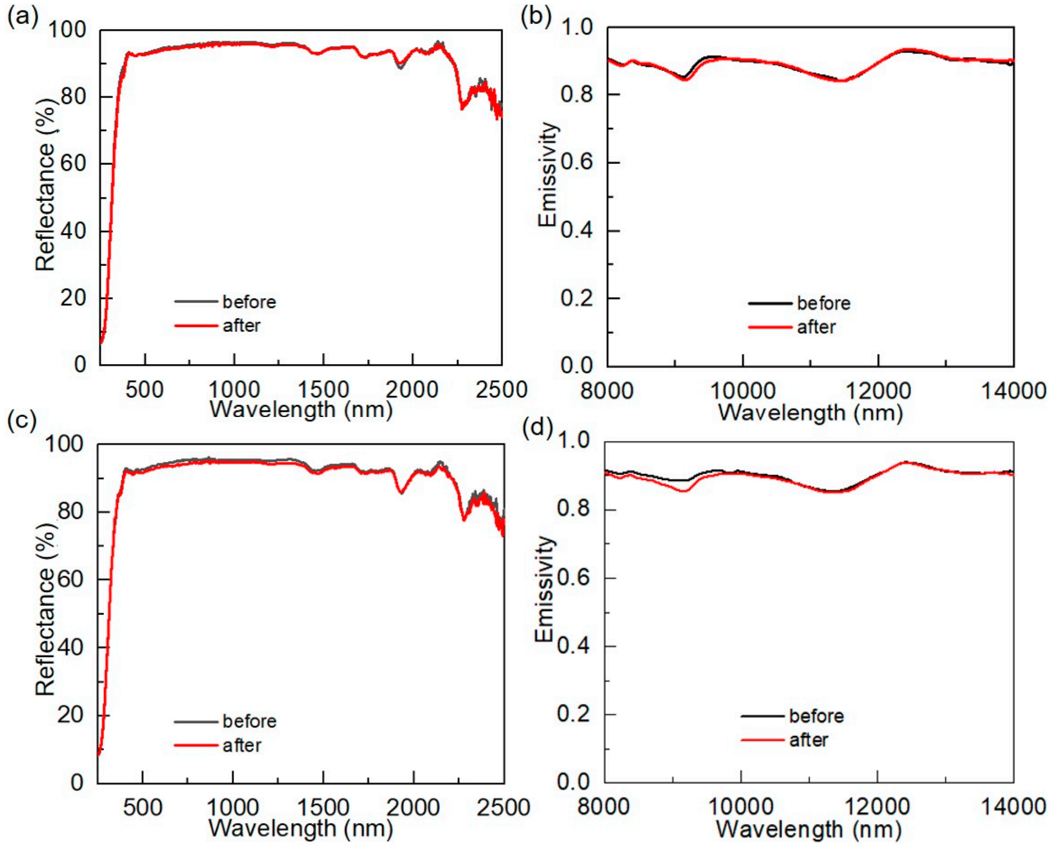

Figure S10. The performance of the coatings before/after the UV accelerated aging test combined with dry–wet cyclic exposure. The reflectance a) and emissivity b) of the aqueous coating. The reflectance c) and emissivity d) of the hydrophobic coating.

Cost of the diatomite-based coating.

Table S2. Cost comparison of diatomite-based solution with other radiative cooling materials.

Reference	Material Category	Unit Price (USD/t)	Amount of Paint Used (kg)	Price (USD/m^2)
This work	Diatomite	300	2.87	0.32
	Methyl cellulose	10000		
	Water	2		
Cheng et al. [28]	$BaSO_4$	350	2	1.8
	SiO_2	850		
	N-methyl pyrrolidone	1200		
	Polyvinylidene Fluoride	3500		
Liu et al. [31]	$BaSO_4$	350	2.35	1.14
	Ethyl cellulose	3000		
	Ethanol	650		
Dong et al. [12]	$BaSO_4$	400		0.5
	$CaCO_3$	150		
	SiO_2	1000		
	Acrylic acid	1200		

The cost of the aqueous coating is listed in Table S2, and the prices of raw materials are based on market research. The cost of our coating is USD 0.32 per square meter. The low cost of our formulation is attributed to several key factors. Firstly, the low density of diatomite, substantially less than that of artificial scatterers like SiO_2 or $BaSO_4$, means less mass is needed to achieve the necessary volume and scattering effect, reducing material costs. Secondly, the use of methyl cellulose as a binder, despite its higher cost, is economically viable due to its high viscosity, which necessitates only a small quantity for effective application. Another key factor in our formulation's cost-efficiency is the use of water as a solvent, in contrast to the expensive organic solvents used in other studies. Since the weight of the paint solution is primarily influenced by its liquid solvent, utilizing water significantly lowers the overall cost of our coating.
